# Roles of miRNAs in Colorectal Cancer: Therapeutic Implications and Clinical Opportunities

**DOI:** 10.34172/apb.2021.029

**Published:** 2020-07-26

**Authors:** Amir Mehrgou, Shima Ebadollahi, Khaled Seidi, Mohammad Hosein Ayoubi-Joshaghani, Amirhossein Ahmadieh Yazdi, Peyman Zare, Mehdi Jaymand, Rana Jahanban-Esfahlan

**Affiliations:** ^1^Department of Medical Genetics and Molecular Biology, Faculty of Medicine, Iran University of Medical Sciences, Tehran, Iran.; ^2^Department of Biochemistry and Biophysics, Faculty of Medicine, Babol University of Medical Sciences, Babol, Iran.; ^3^Biotechnology Research Center, Tabriz University of Medical Sciences, 9841 Tabriz, Iran.; ^4^Drug Applied Research Center, Tabriz University of Medical Sciences, 9841 Tabriz, Iran.; ^5^Student Research Committees, Tabriz University of Medical Sciences, 9841 Tabriz, Iran.; ^6^Research Center for Molecular Medicine, Hamadan University of Medical sciences, Hamadan, Iran.; ^7^Dioscuri Center of Chromatin Biology and Epigenomics, Nencki Institute of Experimental Biology, Polish Academy of Sciences, Warsaw, Poland.; ^8^Faculty of Medicine, Cardinal Stefan Wyszyński University in Warsaw, 01-938 Warsaw, Poland.; ^9^Nano Drug Delivery Research Center, Health Technology Institute, Kermanshah University of Medical Sciences, Kermanshah, Iran.; ^10^Stem Cell Research Center, Tabriz University of Medical Sciences, 9841 Tabriz, Iran.; ^11^Department of Medical Biotechnology, Faculty of Advanced Medical Sciences, Tabriz University of Medical Sciences, Tabriz, Iran.

**Keywords:** Colorectal cancer, miRNAs, Clinical implication, Radiotherapy, Chemotherapy

## Abstract

Colorectal cancer (CRC) is one of the most disseminated diseases across the globe engaging the digestive system. Various therapeutic methods from traditional to the state-of-the-art ones have been applied in CRC patients, however, the attempts have been unfortunate to lead to a definite cure. MiRNAs are a smart group of non-coding RNAs having the capabilities of regulating and controlling coding genes. By utilizing this stock-in-trade biomolecules, not only disease’s symptoms can be eliminated, there may also be a good chance for the complete cure of the disease in the near future. Herein, we provide a comprehensive review delineating the therapeutic relationship between miRNAs and CRC. To this, various clinical aspects of miRNAs which act as a tumor suppressor and/or an oncogene, their underlying cellular processes and clinical outcomes, and, in particular, their effects and expression level changes in patients treated with chemo- and radiotherapy are discussed. Finally, based on the results deducted from scientific research studies, therapeutic opportunities based on targeting/utilizing miRNAs in the preclinical as well as clinical settings are highlighted.

## Introduction

Colorectal cancer (CRC) is one of the most prevalent gastrointestinal malignancies across the world, with over 1.2 million newly reported cases of this disease each year. CRC ranks the third frequent cancer in the globe and is a key cause of cancer-attributed mortality. Moreover, CRC is the second and third most common cancer among women and men, respectively.^1^ Despite all of the medical breakthroughs in the early diagnosis and treatment of this cancer, almost 50% of patients pass away in the 5-year period following the diagnosis due to the metastasis and relapse caused by resistance to treatment. In this regard, one of the primary reasons is that most patients are not diagnosed in the early stages of the disease, and as a result of poor prognosis they would not meet a satisfactory clinical outcome.^2^

CRC is often caused by the accumulation of genetic/epigenetic changes in genes coding for tumor suppressors, oncogenes, and DNA repair pathways. Further, these mutations drive oncogene activation as well as the silencing of tumor suppressor genes. These alterations lead to several changes in cellular processes such as cell proliferation, apoptosis, invasion, and metastasis. The eventual result is the transformation of epithelial cells into adenocarcinoma. Meanwhile, metastasis and drug resistance are among the most common causes of CRC mortality. In metastasis, cancer cells acquire the appearance and the properties of mesenchymal cells by a phenomenon known as epithelial-mesenchymal transition (EMT) which separates them from the primary tumor tissue.^3^ Thereafter, these cells reach the secondary target tissue by overcoming the physiological barriers. Metastatic cells can cross physiological barriers through steps of (*i*) local invasion, (*ii*) intravasation (the invasion of cancerous cells through the basal membrane into blood or lymphatic vessels, which facilitates the migration of cancerous cells from their primary sites), (*iii*) survival in the circulatory system, and (*iv*) extravasation. The cancerous cells enter organs from blood or lymphatic circulations through extravasation and (*v*) colonization in the secondary target tissue. Finally (*vi*), metastatic cells undergo a phenomenon called mesenchymal-epithelial transition (MET) to create a new tumor mass. Therefore, MET process, in which the cells obtain the appearance and the properties of epithelial cells, is the opposite of the EMT process.^4^

Regarding drug resistance, though the process has not been fully uncovered yet, it is suggested that it could be caused by the stem- and stem-like cells. Since metastasis is one of the most important causes of death in CRC patients, the detection of its molecular mechanisms would give a more in-depth insight into CRC prevention and treatment. According to different studies, two of the effective primary treatments for CRC are surgery and chemotherapy. A particular example of surgical treatments is radical surgery, which entails the removal of the blood supply, lymph nodes, and sometimes parts of the structures adjacent to the tumor or cancer tissues. Nevertheless, only 50% of patients with CRC benefit from this treatment while the other 50% experience relapse or tumor metastasis to the other parts of the body. Chemotherapy is another major, and the most common, treatment for CRC. Chemotherapy can be classified into two categories: administration of traditional treatments with unknown cytotoxic effects, and the use of inhibitors of specific molecular pathways in cancer cells. Examples of chemotherapeutic agents for CRC are 5-FU (fluorouracil) and alkylating agents such as oxaliplatin. However, the clinical outcome of this treatment is not satisfactory in all CRC cases due to the development of resistance to chemical agents and the lack of a specific mechanism for diagnosing patients who will definitely benefit from chemotherapy. Hence, there is a considerable need for noninvasive markers for the diagnosis and treatment of cancers, especially CRC with a high morbidity and mortality rate.

### miRNAs biogenesis, origin and function in cancer 

MiRNAs are a large family of single-stranded, short, non-coding endogenous RNAs with a length of approximately 21 to 23 nucleotides. They are generated through the multi-phase biogenesis of the miRNA duplex complex, which itself is produced through the processing of pre-miRNAs using the RNase III Dicer enzyme in endogenous hairpin transcriptions.

Pri-miRNAs are also broken down into 70-85 nucleotide pre-miRNAs and are then cut into temporary double-stranded mRNAs in the cytoplasm. One of the strands carries a mature mRNA sequence with 22 nucleotides in length, and the other is called the complementary strand. These two strands are separated by the microRNP (miRNA plus ribonucleoprotein) complex. During this process, one strand is transformed into an active miRNA and the rest of the strands are decomposed. MiRNAs are hardly degraded due to their small sizes, and are more stable and resistant to chemical, physical, and environmental pressures than the messenger RNAs. A specific region with a length of 2 to 8 nucleotides called the seed sequence at the 5´ end of miRNA sequences, is essential for specific base pairing with the target mRNA molecules. Therefore, miRNAs suppress the expression of 30% of the protein-coding genes by forming incomplete hybrids with specific sites in the 3´UTR regions in the messenger RNA, influencing the mRNA stability. The general mechanism of miRNAs in the animal and mammal cells could be framed through two pathways. One mechanism involves the inhibition of translation following the formation of a complex similar to the RISC (RNA-interference-induced silencing complex). The other mechanism affects the expression of the mRNA-splicing gene.^5^

**Figure 1:**
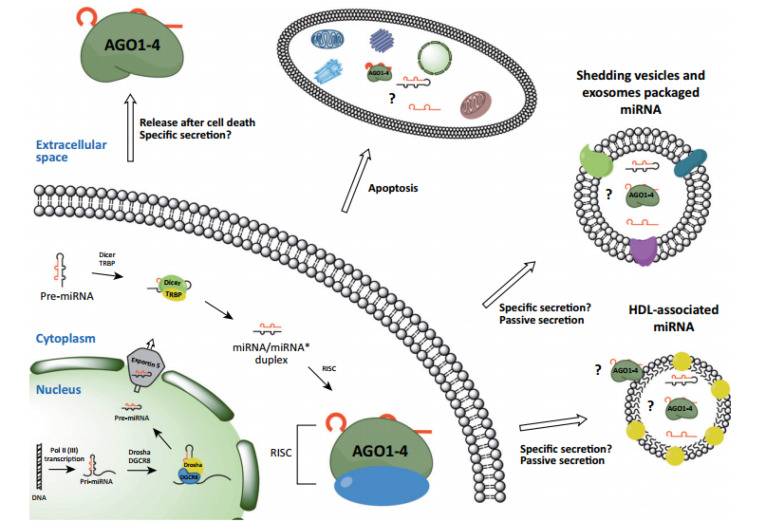
MiRNA biogenesis and the mechanism of miRNA biogenesis in a cell: The RNA polymerase II enzyme transcribes miRNA-encoding genes and produces the primary miRNA (Pri-miRNA) molecules. The Pri-miRNA molecules are converted into a hairpin-structured precursor “Pre-miRNA” molecule by the endoribonucleases DROSHA and DGCR8 (DiGeorge syndrome critical region 8). These processes occur entirely in the nucleus. Then, pre-miRNA is exported to the cytoplasm through a nuclear export receptor called “Exportin 5/RanGTP complex”. In the cytoplasm, this precursor is cleaved by RNase III endonuclease Dicer (Dicer-TRBP) to produce a double-stranded molecule known as a mature miRNA duplex. One of these two strands derived from the mature miRNA duplex incorporation with Argonaute (AGO) proteins family (AGO1-4 in humans) creates RISC (miRNA-induced silencing complex). RISC complex engages in targeting mRNA to mediate gene silencing either by translational repression or by mRNA degradation/deadenylation, modulating the target downstream genes by translational repression or by mRNA degradation/deadenylation. miRNAs can remain and act on the original cell/tissue or can be released into the circulation as free miRNAs (e.g. with AGO proteins or incorporated into HDL particles), or packed in vesicles including exosomes, apoptotic bodies, or shedding vesicles. Reprinted from urchinovich A, Weiz L, Burwinkel B. Extracellular miRNAs: the mystery of their origin and function. Trends Biochem Sci 37:460-5, Copyright (2012) with permission from Elsevier.^7^

Based on their origin, the new classification for miRNAs follows the (*i*) cellular, and (*ii*) cell-free circulatory miRNAs. Accordingly, RNAs can be extracted from cells/tissues (cellular miRNAs), as well as from bodily liquids such as urine, serum, and plasma (circulatory miRNAs). Mature miRNAs can be secreted from the original cell into the circulation as free, in complex with other proteins/lipids, or being packaged into the extra vesicular particles known as exosomes.^4^ Monitoring levels of cell-free miRNAs has emerged as a valuable prognostic and diagnostic tool in cancer (Figure 1).

The malfunction and ectopic expression of the miRNAs profile in many forms of cancer could be attributed to a number of mechanisms such as deletion in the fragile regions of the gene, where more than 52% of miRNA genes which are linked to cancer suppression, are accumulated. This malfunction might also be related to hereditary or acquired mutations in miRNA genes or methylation in the miRNA promoters. Targeting miRNAs as clinical biomarkers may represent a strategy for increasing specificity in overcoming drug resistance.^6^

MiRNAs are extremely protected in all species and play important roles as the translational controllers in vertebrae, worms, and plants. These small molecules are involved in a wide range of cellular and biological processes in CRC, such as inflammation, stress response, migration, cellular differentiation, cell cycle progression, apoptosis, metabolism, changes in extracellular matrix regulators, MET transcription factors, and the onset/progression of cancer. They can also affect tumor survival as well as pathophysiologic processes such as chemoresistance, chemosensitivity, and resistance to radiotherapy.

### The variations of miRNA expressions in CRC

The performance of each miRNA is considerably determined by the cellular and tissue environments. Dysregulated expression of miRNAs is demonstrated in different malignancies including CRC. MiRNAs can serve as oncogenes and/or tumor suppressors in patients suffering from cancers such as CRC depending on the tissue context. A selection of these miRNAs is listed in Table 1 and Table 2, based upon their expression changes and functions. Molecular pathways and their implication in CRC pathogenesis and therapy outcome are discussed in detail in the next sections.

**Table 1:** MiRNA as tumor suppressors in CRC

**MiRNAs as Tumor Suppressor**	**Function in CRC**	**Ref.**
Mir-1	Suppress EMT transition and metastasis via the MAPK and PI3K/AKT pathway	^8^
MIR-9	modulates CRC cell proliferation and apoptosis by regulation of UHRF1	^9,10^
Mir-10b*	Suppresses growth and metastasis by targeting FGF13	^11^
Let-7	Reduce levels of oncoproteins MYC, HMGA2, and IGF1	^12^
Mir-15a	Reduce BCL2 and SOX2 to inhibit tumor growth	^13^
Mir-22	Increase 5-FU sensitivity by promoting apoptosis and inhibiting autophagy in CRC cells	^14^
Mir-27a	Elicits tumor-suppressive effects by targeting SGPP1 and Smad2	^15^
MiR-27a-3p	Controls apoptosis and proliferation via targeting BTG1	^16^
Mir-29c	mediates EMT transition during metastasis via regulation of β-catenin/PTP4A and GNA13 signaling	^17^
Mir-30a	Suppresses migration and invasion by reducing PIK3CD	^18^
Mir-30a	Target insulin receptor substrate 2	^19^
MiR-30a-5p	Suppresses metastasis by targeting ITGB3	^20^
Mir-30b	Targets KRAS, PIK3CD, and BCL2	^21^
Mir-31	Inhibits autophagy in cancer-associated fibroblasts, increase the radiosensitivity and inhibit cancer growth	^22^
Mir-34a	Modulates E2F pathway to induce senescence-like growth arrest	^23^
Mir-34a	suppresses the invasion and migration by enhancing EGR1 and inhibiting vimentin	^24^
Mir-124	Inhibit STAT3 to suppress the growth of human CRC	^25^
Mir-139-5p	Exerts tumor suppressor function by targeting NOTCH1	^26^
Mir-143	inhibit cell invasion and migration via targets MACC1	^27^
Mir-152	Acts as a tumor suppressor gene by targeting	^28^
Mir-203	Targets EIF5A2 to suppress cell proliferation, migration, and invasion	^29^
Mir-203	Suppresses ZNF217 upregulation in CRC and its oncogenicity	^30^
Mir-218	Target the PI3K/Akt/mTOR signaling to inhibit the invasion and migration of colon cancer cells	^31^
Mir-218	Targets cFLIP to promote apoptosis	^32^
Mir-363-3p	Suppresses the EMT transition and inhibits metastasis by targeting Sox4	^33^
Mir-519b-3p	Modulates the uMtCK/Wnt signaling pathway to inhibit the proliferation and invasion	^34^
Mir-873	Targets ELK1 and STRN4 to inhibit metastasis	^35^
MiR-1258	Suppresses CKS1B expression to inhibit the proliferation and migration	^36^

*miRNA with oncogenic/tumor-suppressive dual-action

**Table 2:** Oncomirs involved in CRC pathogenesis

**MiRNAs as Tumor Suppressor**	**Function in CRC**	**Ref.**
Mir-10b*	TWIST-1 induces upregulation of miR-10b and reduce E-cadherin to promote metastatic phenotype acquisition	^37^
MIR-9, mir-31, mir-182	Promote proliferation and tumor cell survival	^10^
Mir-17	promotes cell proliferation, tumor growth, and cell cycle progression by targeting the RND3 tumor suppressor gene	^38^
Mir-23a	Its inhibition activates APAF-1/caspase-9 apoptotic pathway to promotes 5-FU chemosensitivity	^39^
Mir-23b	Its expression regulates EMT transition and causes oxaliplatin-resistant	^40^
MiR-106b	induces cell radioresistance via the activating PI3K/AKT, decreasing PTEN and p21 and upregulation of stemness-related genes (CD133, Sox2)	^41^
Mir-124	Targets iASPP to regulates the cells proliferation	^42^
Mir-125b, mir-137	Its expression upregulates in response to capecitabine chemoradiotherapy which causes drug resistance of rectal cancer	^43^
Mir-135a	Promotes growth and invasion via metastasis suppressor 1	^44^
Mir-135b	Causes proliferation, invasion, and apoptosis by deregulation of PTEN/PI3K pathway, and upregulation of SRC	^45^
Mir-153	Produce MMP9 to promote invasiveness and inhibits FOXO3a to cause drug resistance	^46^
Mir-181b	miR-181b functions as an oncomiR in CRC by targeting PDCD4	^47^
Mir-224	promotes tumor growth and cell proliferation by accelerating the G1-S phase transition via AKT/FOXO3a signaling activation, p21Cip1/ p27Kip1 downregulation, cyclin D1 upregulation, and PHLPP1 and PHLPP2 repression	^48^
miR-501-3p	Promotes progression by activation of Wnt/β catenin signaling	^49^

*miRNA with oncogenic/tumor-suppressive dual-action.

### The Effect of miRNAs on cellular processes involved in CRC development, progression, and drug resistance

MiRNAs and their expression regulation is essential in several human diseases, and cancer in particular, as their involvement in various tumorigenesis processes such as angiogenesis, migration, proliferation, and EMT has been confirmed.^17,38,50,51^

The exogenous expression of Mir-1 suppresses invasive attributes such as migration, proliferation, growth, and metastasis of CRC cells *in vitro*. Mir-1 inhibits metastasis in CRC cases by targeting LASP1. In addition, mir-1 prevents tumor progression through dephosphorylation of ERK1/2 and AKT in the MAPK/ERK and PI3K/AKT pathways. Mir-1 also suppresses EMT and increases MET by reducing mesenchymal markers such as fibronectin and elevating the epithelial markers such as E-cadherin.^8^ Mir-7 regulates cell proliferation *in vitro*.^6^ Mir-9 is transcribed from the mir-9-1/9-2, 9-3 genes, and the CpG island methylation in mir-9-1 is believed to be associated with lymphatic metastasis in CRC samples and cell lines.^52^

Mir-9 is shown to act as a tumor suppressor gene, as its increased expression is shown to correlate with the decreased expression of UHRF1, which is involved in DNA methylation, and cell proliferation in CRC tissue samples. Meanwhile, mir-9 expression can counteract UHRF1 function, and promotes the apoptosis of CRC cells *in vitro*.^9^

Example of miRNA with dual action is MiRNA-873. While it acts as an oncogene to derive lung adenocarcinoma cell migration and proliferation by targeting SRC kinase signaling inhibitor 1,^53^ its expression shows a decreased level in mouse CRC samples, human CRC clinical specimens, and highly metastatic CRC cell lines. Mechanistically, MiRNA-873 suppresses CRC metastasis by targeting ELK1-Cyclin D1 pathway, STRN4, and promoting EMT process *in vitro* and *in vivo*.^35^

Another important CRC-related MiRNA is Mir-17, which increases cell growth, proliferation,^38^ and cell cycle transition from phase G0 to G1 as well as from phase G1 to S by suppressing the RDN3 tumor suppressor gene. RND3 is involved in the adenoma to carcinoma transition in CRC patients. Moreover, mir-17-5p is known to be linked to metastasis and the clinical stage of CRC.^38,54^

Likewise, Mir-21 can reduce apoptosis, increase the invasion depth, and increase lymphatic metastasis by inhibiting PTEN. It is also associated with the enhanced cell proliferation and advanced stage of TNM clinical classification.^51,55^

Mir-21 induces stem cell properties in CRC cells. Mir-21 also promotes cell proliferation, and decreases sensitivity to the clinical regimens that are based on 5-FU and oxaliplatin in the HT-29 and HCT116 cell lines.^56^ Additional miRNA linked with stem cell-like properties is Mir-145. It regulates the renewal property of stem cells and diminishes the pluripotency factors such as Oct4, Sox2, and Nanog through which it inhibits the stemness features of cells. Mir-145 prompts growth and differentiation of HT-29 and HCT-116 cancer cells *in vitro*. In a mutual inverse relationship with mir-21, mir-145 regulates the differentiation and proliferation of stem cells, and the growth of cancer stem cell (CSC)-enriched colon spheres and contributes to the regulation of chemoresistance, relapse, and metastasis.^56^

By inhibiting TIAM1, MMP2, and MMP9, mir-22 inhibits cell motility and metastasis in CRC,^57^ and also promotes the increased apoptosis in the SW620 cells by promoting the caspase-9 and caspase-7 apoptotic proteins and PARP. Moreover, mir-22 inhibits autophagy in SW620 and RKO cells by reducing the expression of autophagy marker LC3-II which increases their sensitivity to 5-FU treatment *in vitro* and *in vivo.* Similar to the actions of mir-22 which can counteract the action of autophagy inhibitors by targeting B-cell translocation gene 1 (BTG1),^14^ recently miR-27a-3p depletion has also been shown to increase apoptosis and reduce proliferation of colon cancer cells by upregulating BTG1 and suppressing ERK/MEK signaling pathway.^16^

In contrast, Mir-23a expression is shown to suppresses the apoptosis induced by 5-FU through the APAF1/caspase9 pathway in the HCT-116 and HT-29 cell lines.^39^ Mir-27a can block cell proliferation, colonization, and growth of CRC tumors by targeting SGPP1, Smad2, and STAT3. The upregulation of mir-27a is associated with distant metastasis and histopathologic stages.^15^

Mir-29c blocks metastasis and invasion in CRC by diminishing the expression of GNA13 and PTP4A1 genes and suppressing the β-catenin pathway. Mir-29c also limits cell migration by increasing epithelial markers such as E-cadherin and β-catenin and reducing mesenchymal markers such as fibronectin and vimentin which cause MET-related morphologic changes.^17^ Mir-29c-3p is a critical target of the P53 gene and regulates the expression of Pleckstrin Homology-Like Domain Family Member 2 (PHLDB2), which is involved in colon cancer metastasis in CRC patients.^58^

Mir-30a is known to prevent various pathways in CRC including migration, invasion, cell growth, cell proliferation, and metastasis. This action is mediated by reduced phosphorylation of akt by targeting insulin receptor substrate 2 *in vitro*.^19^ Another important target of mir-30a is the Transmembrane-4-L-six-family-1 (TM4SF1), the up-regulation of which in CRC tumor specimens is shown to be associated with advanced stage and lymph node metastasis. Mir 30a expression suppresses invasion of CRC cancer cells by targeting TM4SF1 as well as VEGF and E-cadherin as EMT regulators.^59^ In addition, miR-30a-5p is found to inhibit colon metastasis through restraining integrin β3.^20^

As another member of mir-30 family, Mir-30b inhibits cell proliferation and tumor growth by enhancing the cell population in the G0/G1 phase and inhibiting transition from G2/M and S phases. It also increases apoptosis by suppressing BCL-2 activity, KRAS, PIK3CD.^21^

Furthermore, suppression of mir-31 expression diminishes cell migration and invasion. The increased expression of mir-31 in the CRC HT29 cell line, which carries the TP53 mutation, exerts a strong anti-apoptotic effect. However, the anti-apoptotic effect of increased MIR-31 expression in SW480 and HCT116, which have an intact and functional TP53 pathway, is weaker or absent.^10^

MiR-103/107 is overexpressed in CRC where it leads to elevated metastasis by targeting metastatic suppressors such as death-associated protein kinase and Krüppel-like factor 4.^60^ Equally, Mir-106b enhances cell proliferation and tumor growth *in vitro* and * in vivo*. It also increases resistance to radiation by functioning as a DNA repair agent, and reduces apoptosis by promoting BCL2 expression. When mir-106b expression is increased, p21 acts as a DNA damaging agent in SW620 cells and reverses resistance to radiation, which also involves PTEN. Moreover, mir-106b promotes the self-renewal and stemness properties in cells under both normal and radiated conditions.^41^

Mir-124 increases apoptosis and diminishes tumor growth *in vitro* and *in vivo* by targeting STAT3 and inhibitor of apoptosis-stimulating protein of p53 (*iASPP*), and limits cell proliferation and colonization in the CRC cell lines.^25,42^ Mir-125b is essential for the continued proliferation of differentiated cells, while both mir-125a and mir-125b decrease migration and invasion in rectal cancer.^43^ Moreover, in vitro investigations have recognized that mir-125b elicits an oncogenic role in the therapeutic resistance and progression in CRC by inhibiting critical proteins such as P53 and BAK1 (Bcl2L7).^61,62^

Mir-129 enhances apoptosis by suppression of Bcl-2, and by blocking 5-FU-targeted proteins such as TS and E2F3, eventually stopping cell growth and cell cycle progression in CRC patients.^63^ Mir-130b restrains cell invasion and migration in CRC by suppressing integrin β1.^50^ Furthermore, Mir-135a increases cell proliferation in the SW480 and SW620 cell lines by metastasis suppressor 1.^44^ Mir-135b promotes proliferation, invasion, migration, and neo-angiogenesis in CRC patients by elevating IL-8.^45^ Low levels of Mir-137 are required for the maintenance of the tumor state in rectal cancer.^43^ By targeting IGF-1R, mir-139 reduces invasion, migration, and metastasis *in vitro* and *in vivo*. Mir-139 expression is linked to the tumor stage, the involvement of lymph nodes, and vascular invasion.^64^

Mir-139-5p can promote cell cycle arrest in phase G0/G1 via the upregulation of p21Cip1/Waf1and p27Kip1. It also escalates apoptosis through the extrinsic apoptosis pathway, including the caspase-3, caspase-7, and caspase-8 proteins and the cleaved PARP. Notch is an important target of this miRNA, as well. Furthermore, mir-139-59 plays a role in the inhibition of invasion and migration by reducing matrix metalloproteinases (MMP7) and MMP9 in mice.^26^

Moreover, Mir-143 prevents cell growth by suppressing oncogenic proteins such as KRAS, metastasis-associated in colon cancer-1 (MACC1), C-myc, and IRS-1, and can limit invasion and metastasis in CRC.^27,65,66^ Downregulation of mir-143 is linked to distant metastasis and tumor relapse in CRC patients.^65^ Mir-150 inhibits tumor growth *in vitro* and *in vivo* by inhibiting DKC1 and AKT2, and increasing tumor suppressors such as Bim and P53 in vivo.^67^ Mir-153 promotes invasion in CRC by increasing MMP-9, which is a key destructor of the extracellular matrix and is significantly involved in CRC invasion. Moreover, mir-153 reduces apoptosis and increases resistance to chemotherapy by decreasing caspase-3 in the SW480 cell line treated with cisplatin. Also, miR-153 has been observed to be up-regulated in CRC, leading to elevated invasion.^46^ Mir-155 is linked to lymphatic metastasis and promotes invasion and migration by targeting claudin-1. Mir-155 also serves as an EMT mediator by inhibiting E-cadherin and increasing ZEB1.^68^ Mir-155 together with mir-193, target RAD51 which is involved in DNA repair.^69^

The overexpression of miR-181b through direct binding to tumor suppressor RASSF1A boosts its expression in CRC cells.^70^ On the other hand, miR-181b has the most potential pre-proliferation effect on the HT-29 cell line, which increases cell proliferation by more than four times.^10^

Mir-15B and Mir-182 elicit anti-apoptotic properties. Mir-182 promotes progression of CRC by increasing cell survival.^10^ The increased expression of MIR-185 and the decreased expression of MIR-133B are linked with the development of metastatic conditions in CRC cases.^71^ Mir-200c, mir-212, and mir-363-3p have been recognized as EMT regulators. Anti-metastatic potential is attributed to targeting ZEB1, ETS1 and FLT1 and EMT markers E-cadherin and vimentin by mir-200c,^72^ superoxide dismutase (MnSOD) targeting by mir-212 ^73^ and Sox-4 targeting by mir-363-3p in CRC in tissue specimens, in vitro and in vivo.^33^

The expression of miRNAs such as miR-200,^74^ miR-135,^75^ and miR-34a^23,24^ regulate cell survival, proliferation, invasion, and metastasis in CRC. A recent study indicated that mir-34a inhibited the invasion and migration of SW620 cells by blocking vimentin and increasing early growth response protein 1 (EGR1).^24^ MiRNA-1258 elicits tumor-suppressive effects and suppresses the proliferation and migration of human CRC Cells by inhibiting cyclin-dependent kinase regulatory subunit 1B (CKS1B).^36^ Also, miR-501-3p promotes CRC progression by activation of Wnt/β-catenin signaling and through reducing expression of their target genes, cyclin D1 and c-Myc *in vitro*.^49^

Mir-203 reduces cell proliferation and growth and increases apoptosis and cellular senescence. It also causes oxaliplatin resistance of CRC cells by downregulating DNA damage response mediators, ataxia telangiectasia mutated (ATM) kinases.^76^ The increased mir-218 expression precludes the growth, motility, and invasion of LoVo CRC cells.^31^

Mir-218 induces the apoptosis of SW1417 cells by increasing caspase-8 expression and inhibiting cellular c-Fas-associated death domain-like interleukin-1β-converting (c-FLIP) regulator.^32^ Mir-222 increases cell proliferation and migration and reduces apoptosis.^77^ Mir-223 improves the cellular proliferation of CRC cells as well as their migration and invasion capabilities.^78^ Mir-224 increases cell proliferation and tumorigenesis by suppressing two tumor suppressors named PHLPP1 and PHLPP2* in vitro* and *in vivo*. In addition, mir-224 promotes cell survival.^48^

Dysregulated mir-224 expression is associated with declined chemoradiosensitivity in CRC. Therefore, mir-224 has pro-metastatic characteristics rather than anti-metastatic role in CRC.^79^ Mir-297 induces sensitivity to several chemotherapeutics *in vitro* and *in vivo*.^80^ Mir-378 constrains cell growth in the transition from phase G1 to S, and decreases cell life and colonization in the HT-29 and HCT-116 cell lines.^81^

MIR-519 reduces cell proliferation by controlling the levels of RNA-binding protein HUR. HUR regulates the translational efficiency and stability of mRNAs encoding key elements of the cellular growth, proliferation, and survival pathways. HUR also uses alternative polyadenylation sites to modulate its own expression.^82^

### miRNAs expression changes determine treatment outcomes

#### Chemotherapy

It is well accepted that miRNAs confer strong regulatory effects on survival rate by regulating the pathways for chemical resistance and sensitivity.^46^

In addition, the existing pieces of evidence suggest that changes in the expression of miRNAs are in fact responses to chemotherapeutic agents. These changes, which are made during chemotherapeutic regimens such as 5-FU, have been observed in the human CRC cell lines.^66^ Mir-7 is capable of suppressing EGRF *in vitro* and making CRC patients, who develop resistance to EGFR inhibitors such as cetuximab, regain sensitivity to these drugs.^6^

For more than 50 years, advanced CRC patients have been using fluoropyrimidine-based chemotherapeutic agents such as 5-FU.^63^ Nishida et al. found that the expression of miR-10b is a strong indicator for measuring the sensitivity to the conventional chemotherapy regimens based on 5-FU.^83^ Mir-17-5p results in chemoresistance in CRC patients by inhibiting the PTEN gene through binding to two regions in the 3’UTR region of this gene. Hence, the MIR-17-5p antisense oligonucleotide can be a promising solution for overcoming chemoresistance in these patients.^54^ MIR-21 is one of the most significant miRNAs with oncogenic activity in CRC. This miRNA exerts its effect by constraining the clinical effect of 5-FU-based chemotherapeutic regimens.^56^ In addition, mir-21 is downregulated in the CRC cell lines treated with 5-FU.^66^

From another angle, Mir-22 potentiates chemosensitivity to paclitaxel. Particularly, it diminishes chemoresistance to this drug in CRC patients who carry mutated P53 through the PTEN signaling pathway. MIR-22, through suppressing the target enzyme of 5-FU (Thymidylate synthase (TS)), escalates the apoptosis induced by this medicine in SW620 cells and elevates the sensitivity of CRC cells to 5-FU. Moreover, treatment with 5-FU upregulates MIR-22 in RKO cells and facilitates apoptosis. Equally, the inhibition of MIR-22 also reduces the 5-FU-related apoptosis in RKO cells. Furthermore, MIR-22 can sensitize cells to 5-FU by preventing autophagy. Based on the results from the *in vivo* experiments, MIR-22 reduces the average weight of CRC tumors by elevating chemosensitivity to 5-FU.^14^ In the condition of 5-FU treatment, the expression of mir-23 in C.20.22 and HC.21 CRC cells, and mir-23a expression in HT29 and HCT.116 colon carcinoma cells, are escalated. The increase in apoptosis induced by 5-FU through the in vitro administration of mir-23a antisense supports the finding that mir-23 functions as a 5-FU-based drug resistance agent.^39^ Mir-23a and mir-497 have been recognized as regulators in 5-FU treatment. Mir-497, miR-139-5p, and mir-23a sensitize CRC cells to 5-FU by serving as tumor suppressors.^84,85^ The suppression of mir-31 declines the resistance to 5-FU in HCT116 cells.^10^ Mir-129 leads CRC cells to be sensitized to 5-FU *in vitro* and *in vivo* in two ways; either by (*i*) downregulating Bcl-2 expression, which leads to high apoptosis rate; or by (*ii*) suppressing the E2F3 protein.^63^ Mir-139-5p sensitizes CRC cells to 5-FU through inducing apoptosis and inhibiting the expression of miR-139-5p targets NOTCH-1 gene and its downstream MDR-associated genes, MRP-1, and Bcl-2 .^85^

Mir-140 creates resistance to the 5-FU in CRC patients.^86^ Also, the downregulation of mir-148a reduces sensitivity to 5-FU and oxaliplatin.^87^ A high level of miR-625-3p declines the response to oxaliplatin in patients suffering from metastatic CRC.^88^ The upregulation of mir-153 enhances resistance to platinum-based treatments such as oxaliplatin and cisplatin through inhibiting FOXO3. This miRNA reduces the apoptotic effect of these drugs.^46^ According to the findings reported by Nakajima et al, let-7 and miR-181b are linked to the response to 5-FU treatment.^89^ MiR-203 influences the response of CRC cells to 5-FU by inhibiting TYMS, and inhibits tumor growth.^90^ It also positively regulates the CRC cell responses to paclitaxel, which affects the cell cycle, by reducing AKT. Mir-203 induces cellular senescence and causes delayed cell growth. However, miR-203 can contribute to the increase in the acquired resistance to oxaliplatin in CRC cells.^76^

Mir-19b, miR-34, and miR-192/215 are other miRNAs determining the resistance to 5-FU.^91-93^ Some of the miRNAs linked to the oxaliplatin resistance are miR-20 and miR-1915.^94,95^ Mir-433 sensitizes HeLa cell to 5-FU by reducing TYMS expression, and is downregulated in CRC tissues and cell lines. MiR-433 targets MACC1 to promote apoptosis and decrease the viability of CRC cells.^96^

Svoboda et al. reported that let-7e, miR-99a, miR-215, miR-450b-5p, and miR-196b are involved in the CRC response to chemotherapy.^97^ It was reported that mir-99 impedes pro-survival/anti-apoptotic pathways through affecting Bcl-2 and mTOR targets.^98^ The upregulation of miR-592 has been observed in CRC patients with a healthy MMR (Mismatch Repair) system as compared to patients with defective MMR system. Moreover, cells with defective MMRs and a reduced level of miR-592 are resistant to some chemotherapeutic agents such as 5-FU and some platinum compounds such as cisplatin and carboplatin.^86,99^ MiR-127 expression is decreased in doxorubicin-insensitive colon cancer cell lines.^100^ MiR-127 is shown to inhibit proto-oncogene Bcl-6 activity.^101^ Lower levels of miR-150 in CRC tissue samples correlates with chemoresistance and shorter survival.^67^

#### Radiation therapy

Intrinsic and acquired resistance to radiation is a challenging barrier to the clinical management of CRC. The acquisition of resistance to radiation is a complex process comprising an increase in the expression of DNA repair proteins, and dysregulation in signaling pathways, angiogenesis, CSCs, and autophagy.^102^ MiRNAs may serve to predict and modify cancer treatments such as radiotherapy.^102^ Recent findings are also indicative of the substantial role of miRNAs in the cellular responses against ionizing radiation.^103^

Lin28-let7 mediates radiation resistance in CRC cancer cells by activating K-Ras.^104^ Moreover, the combined functions of let7g and mir-9 can increase resistance to radiotherapy through the suppression of NF-kappaB1.^105^ MIR-101 sensitizes CRC cells to radiation by targeting ATM and *in vitro* and *in vivo*.^106^ The clonogenic assay is a cell biology technique that determines the effectiveness of specific agents on cell survival and proliferation. This assay revealed that the upregulation of MIR-106b results in an increase in resistance, while its downregulation leads to an increase in sensitivity to ionizing radiation in CRC patients. Therefore, it is concluded that cells with upregulated mir-106b have stronger DNA repair systems. Besides, mir-106b induces this resistance by increasing AKT and reducing PTEN in the PTEN-PI3K/AKT pathway.^41^ MIR-124 increases CRC cells’ sensitivity to ionizing radiation and promotes cell apoptosis in radiotherapy by declining the expression of PRRX1. Moreover, mir-124 suppresses Bcl-2, and the synergy between MIR-124 and ionizing radiation drastically diminishes Bcl-2 expression and eventually leads to apoptosis.^102^

### The effect of miRNAs on CRC clinical responses and procedure 

MiRNAs are implicated in various stages of tumorigenesis, form early CRC development to acquiring drug resistance and metastasis^38^ as well as the survival rate of CRC patients. A high level of let-7 is linked to a shorter lifetime and is contradictory to let-7 role as a tumor suppressor.^86^ The in-situ hybridization results suggest a sharp decrease in the expression of mir-9 in CRC tissues compared to healthy counterparts. Patients experiencing reduced expression of mir-9 have poor clinical outcomes.^9^ MiR-17-5P reduces the survival of CRC patients by deactivating PTEN.^54^ Schetter et al have demonstrated that mir-21 overexpression is linked to poor clinical outcomes and low survival rate in colon adenocarcinoma patients.^107^

MIR-21 also plays a substantial role in the pathogenesis of CRC. Suppression of PTEN is controlled by miR-21, which is associated with reinforcement of the PI3K pathway and the advancement of CRC.^66^

Low expression of miR-30b is linked to low differentiation and the advanced stage of TNM clinical classification. Moreover, patients with upregulated miR-30b survive longer than patients with low expression of this miRNA.^21^ MiR-93 can also prevent the early relapse of CRC.^108^

Research findings have revealed that chaotic mir-106a expression, independent of the tumor stage, can function as a DFS (disease-free survival) and OS (overall survival) marker.^107^ There was no considerable difference in the expressions of miR-129 in the healthy tissues and adenomas in stages I and II of cancer, but miR-129 levels dropped significantly in stages III and IV of cancer, revealing the relationship between reduced expression of mir-129 and CRC progression.^63^ The poor survival of CRC patients is also connected with the declined expression of mir-133B.^71^ Although there are contrasting reports for relation of mir-139-5P with the CRC progression, Zhang et al. reported a decreased level of this miRNA with advanced CRC.^26^ Moreover, expression changes of miR-21, miR-31, miR-143, and miR-145 are closely involved in the pathological and clinical characteristics of CRC.^10,56,109^

The downregulated mir-150 is linked to poor clinical outcomes in patients with stages I and II of CRC. Hence, increased mir-150 activity contributes to the prevention of tumorigenesis in populations prone to CRC, such as people suffering from familial adenomatous polyposis (FAP).^67^ The upregulated mir-153 in advanced CRC is linked to cancer progression and the tumor spreads to distant and nodal organs.^46^ Overexpression of mir-185 is significantly linked with the low survival rate in CRC cases.^71^ Moreover, high expression of mir-29a and mir-362-3p elongates DFS.^110,111^

Mir-451 can be used as a marker for predicting drug resistance (e.g. response to irinotecan) and relapse in CRC cases.^112^ Equally, the elevated levels of miR-3 and reduced expression of mir-592 are linked to poor responses to treatment.^86^

### MiRNAs as biomarkers in CRC patients

MiRNAs are used as novel biomarkers in CRC patients. These smart molecules are used as prognostic and diagnostic biomarkers for the risk assessment of cancer and the analysis of response to treatment, and are even considered as therapeutic agents and targets.^113^ The dysregulation of miRNAs is also involved in the development and progression of CRC.^21^ The declined expression of mir-7 is associated with CRC progression and poor prognosis in CRC patients. Moreover, mir-7 can be considered a useful marker for sensitivity to cetuximab.^6^ Also, the downregulated mir-9 in CRC tissues is linked to CRC prognosis.^114^ High expression of mir-15 is known as a marker for poor prognosis in CRC cases.^115^ Mir-17-5b is known as a biomarker that demonstrates the prognosis and prediction of chemotherapy in CRC cases. This miRNA is particularly associated with the final stage of CRC and a worse survival rate.^54^ The increased expression of mir-21 in CRC tumor tissues is considered as a biomarker for the advanced stage of this disease, and poor cellular differentiation and response to chemotherapy.^116^ Therefore, this miRNA provides a diagnostic value for CRC.^51^ In addition, the expression of mir-31, similar to mir-21, is linked to the disease stage.^10^ Mir-34a may function as a non-invasive biomarker for CRC.^24^ Furthermore, Wang et al. carried out a microarray test to show that the expression of mir-106b is enhanced in CRC cases with lymphatic metastasis.^117^ The dysregulation of mir-139-5p in the early stage of CRC has also been reported.^26^ Several research results have indicated that mir-15b, mir-181b, mir-191, and mir-200c may play a role in the CRC development and its progression, and so they may also be involved as non-invasive markers in the prognosis of this disease.^113^

Mir-218 and miR-152 are linked to the TNM stage of CRC and a decrease in their expression has been observed in patients with poor prognosis.^28,118^ Also, miR-224 promotes cell metastasis and proliferation through the Wnt/β-catenin pathway. These results demonstrate that the increase in the expression of mir-224 is considered to be linked to the severe phenotype of CRC, which includes its invasive characteristics and poor prognosis in patients suffering from CRC.^48,119^

**Figure 2:**
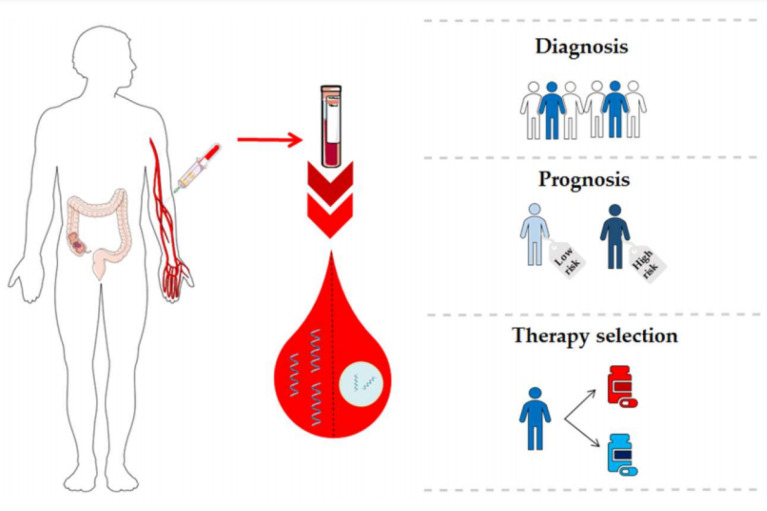
Concept and significance of liquid biopsy using circulatory miRNA for CRC personalized medicine. Adopted from MDPI: *Cancers*.^120^ Copyright (2019).

Importantly, recent advances in single-cell analysis have opened a new horizon in the non-invasive analysis of bodily fluids for the detection of cancer biomarkers, including circulatory miRNAs.^120,121^ This field, which is referred to as “liquid biopsy”, combined with newly-emerged nanobiosensors,^122,123^ and lab-on-chip and microfluidics devices^124^ has enabled researchers and clinicians to analyze low amounts of samples to gain valuable information regarding active and/or non-active disease state (Figure 2, Table 3). Circulatory miRNA with diagnostic/prognostic potential in CRC in recently reviewed by.^120^

**Table 3:** Circulatory miRNA as valuable diagnostic/prognostic in CRC patients

**miRNA**	**Type**	**Expression pattern in CRC**	**Clinical value**	**Ref.**
mir-125b	Plasma exosomes	Increased	Early detection of resistance to first-line chemotherapy (leucovorin, oxaliplatin, fluorouracil) in patients with advanced/recurrent CRC/biomarker for PFS	^125^
Mir-21	Plasma exosomes, primary tumor tissues, liver metastasis tissues	Increased	A prognostic factor for OS rates in patients with TNM stage IV and DFS rates with TNM stage II or III in CRC patients	^126^
miR-125b-2-3p	Tissue	Decreased	Prediction of CRC cell sensitivity to first-line chemotherapy (fluorouracil, oxaliplatin, CPT-11)	^127^
miR-652-3p, miR-328-3p, miR-342-3p, miR-501-3p	Plasma	Increased	Prediction of OS rates and tumor relapse of stage I–III	^128^
miR-652-3p	Serum, plasma, tissue	Increased	Biomarker for chemo (multi-tyrosine kinase inhibitor regorafenib) -refractive metastatic CRC	^129^
Mir-1290	Tissues, serum	Increased	Correlation with tumor aggressiveness and poor prognosis. Independent prognostic factor and an independent predictor for tumor recurrence.	^130^
Mir-122	Serum exosome	Increased	A new diagnostic and prognostic biomarker in CRC patients with liver metastasis	^131^
miR-150-5p	Serum exosome	Decreased	Association with poor differentiation, advanced TNM stage, positive lymph node metastasis and poor survival rates in CRC patients	^132^
miR-1290, miR-320d	Plasma	Increased	Early diagnosis of CRC	^133^
miRNA-320d	Serum exosome	Increased	Diagnostic biomarker for metastatic CRC	^134^
miR-182, miR-20a	Tissue, plasma samples	Increased	Diagnostic biomarker for early CRC	^135^
miR-27a, miR-130a	Serum exosome	Increased	Higher levels of these miRNA can be used for early detection and predicting prognosis of CRC	^136^
miR-103a-3p, miR-18a-5p, miR-127-3p, miR-17-5p	Tissue	Increased	Diagnostic biomarker for early CRC	^137^
miR-17-5p, miR-18b-5p, miR-181a-5p, miR-18a-5p	Plasma exosomes			
miR-19a-3p, miR-21-5p, miR-425-5p	Serum, tissue, serum exosome	Increased	Diagnostic biomarker for early CRC	^138^
miR-92a-3p, miR-17-5p	Serum exosome	Increased	Predicting biomarker for CRC staging and grading	^139^
Mir-200c	Serum	Increased	CRC prognosis and predicting metastasis	^140^
miR-200c-3p, miR-141-3p, miR-143-3p	Tissue, serum	Increased	Differentiation of advanced adenoma from CRC	^109^
Mir-203	Serum	Increased	Prognostic biomarker for distant metastasis, higher tumor stage, poor survival rate	^141^

Abbreviations:OS, overall survival; DFS, disease-free survival; PFS, progression-free survival.

## Conclusion

CRC is one of the prevalent cancers in which the benefits of using miRNAs as a non-invasive diagnostic and prognostic tool can be reaped. In the sense of liquid biopsy, circulatory levels of miRNA are applied as a non-invasive method for screening of CRC. Generally, several hundred miRNAs are observed to be drastically dysregulated in this disease. The miRNAs target a wide range of molecular pathways in normal and cancerous cells. Therefore, this significant feature makes these molecules good candidates for playing a role as biomarkers for differentiating stages of the disease. Also, taking advantage of these smart molecules as therapeutic hallmarks, CRC patients could be detected easier and sooner, meanwhile, the clinical status and the efficiency of clinical interventions for them could be meticulously monitored while they are receiving clinical regimens. However, the specificity and sensitivity of the discussed method are not approved as same as those of routine clinical ones. Accordingly, this method could not be an undisputed substitution for colonoscopy or other commonly-used diagnostic methods, nor for chemotherapy and radiotherapy as standard treatment methods yet, but as a complement to them. Furthermore, technological advances that allow for single-cell analysis of CRC biomarkers such as cell-free miRNA would allow effective screening and predict disease state. Equally it affords clinicians to adopt the most appropriate therapeutic regimes before cancer can progress into an advanced disease.

## Ethical Issues

Not applicable.

## Conflict of interest

The authors declare no conflicts of interest.

## Authors’ contribution

AM and ShE have written the initial draft, KhS and MHAJ collected the data. AhA has drawn the figures. MJ, PZ and RJE have revised, edited and polished the final draft for intellectual content.
